# Supplementary material for: Susceptibility to Degradation in Soil of Branched Polyesterurethane Blends with Polylactide and Starch
Source: Polymers (Basel). 2022 May 20;14(10):2086. doi: 10.3390/polym14102086 (PMC9144702; doi:10.3390/polym14102086)
Supplement: Supplementary file 1 [file polymers-14-02086-s001.zip › polymers-1682077-supplementary.pdf]

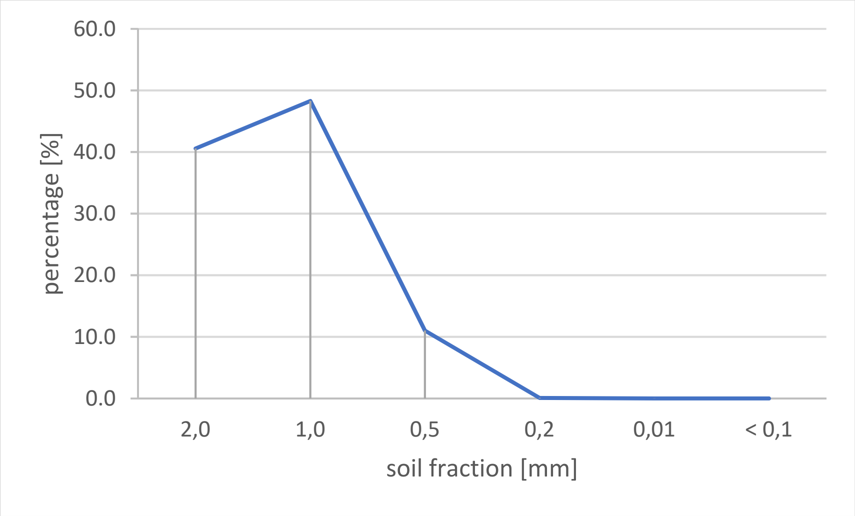

Figure S1. Soil sieve analysis.

Table S1. Basic soil parameters (before and after adding the biohumus solution).

Parameter	Analysis method	Device	Unit	Soil	Soil with biohumus
conductivity	PN-EN 12457-4:2006 oraz PN-EN 27888:1999	inoLab® Cond 7310	µS/cm	154.0	168.1
density of soil solids		biuret method	g/cm^3	2.28	2.14
ammonium nitrogen	PB-178 Hamilton (in-house)	Titration method	% s.m	0.00337	0.00429
Kiejdahl nitrogen	PN_EN 13342:2002	KjelROC Analyzer, KD-310-A	% s.m.	0.20	0.24
DOC (Dissolved Organic Carbon)	PN-EN 12457-4:2006; PN-EN1484:1999	ANALITYCJENA, Multi N/C 3100 N3-1552/AU	mg/kg	100.9	130.5
TOC (Total Organic Carbon)	PN-EN 12457-4:2006; PN-EN1484:1999	ANALITYCJENA, Multi N/C 3100 N3-1552/AU	mg/l	10.09	13.05
Zn	ICP-OES	Thermo Scientific™ iCAP™ 7400 ICP-OES Analyzer	mg/kg	32.228	33.4
Cu	ICP-OES	Thermo Scientific™ iCAP™ 7400 ICP-OES Analyzer	mg/kg	4.33	4.412
Ni	ICP-OES	Thermo Scientific™ iCAP™ 7400 ICP-OES Analyzer	mg/kg	3.006	2.854
Pb	ICP-OES	Thermo Scientific™ iCAP™ 7400 ICP-OES Analyzer	mg/kg	10.31	9.712
Cr	ICP-OES	Thermo Scientific™ iCAP™ 7400 ICP-OES Analyzer	mg/kg	11.448	10.954
Cd	ICP-OES	Thermo Scientific™ iCAP™ 7400 ICP-OES Analyzer	mg/kg	0.285	0.276
Fe	ICP-OES	Thermo Scientific™ iCAP™ 7400 ICP-OES Analyzer	mg/kg	6456.494	6138.294
Ca	ICP-OES	Thermo Scientific™ iCAP™ 7400 ICP-OES Analyzer	mg/kg	3121.167	13196.403
Mg	ICP-OES	Thermo Scientific™ iCAP™ 7400 ICP-OES Analyzer	mg/kg	1272.956	1184.888
P	ICP-OES	Thermo Scientific™ iCAP™ 7400 ICP-OES Analyzer	mg/kg	685.023	664.544

K	ICP-OES	Thermo Scientific™ iCAP™ 7400 ICP-OES Analyzer	mg/kg	1645.226	1673.819
As	ICP-OES	Thermo Scientific™ iCAP™ 7400 ICP-OES Analyzer	mg/kg	2.261	2.612
Hg	CVAAS	MA-3000 NIPPON Instruments Corporation	mg/kg	0.021	0.014

Table S2. Microbial characteristic of soil with biohumus

**Parameter**	**Method**	**Culture medium**	**Number of colony-forming bacterial/fungi units [CFU/g]**
Total bacteria count (TBC)	PN-EN ISO 4833-1:2013-12+Ap1:2016-11, surface plating technique	Tryptone Soya Agar	2.0×10^7
Total Yeast and Mold Counts (TYMC)	PN ISO 7954:1999, surface plating technique	Yeast Extract Glucose Chloramphenicol Medium	3.3×10^5
Escherichia coli	PN-EN ISO 16649-2:2004, pour plate method	Chromid Id Coli agar	<10
Citrobacter spp.	PN-EN ISO 16649-2:2004, pour plate method	Chromid Id Coli agar	6.4×10^2
Salmonella spp.	PN-Z-19000-1:2001, surface plating technique	Brilliance Salmonella Agar	4.6×10^4
Bacillus spp.	PN-EN ISO 7932:1999, pour plate method	Bacillus ChromoSelect Agar	4.4×10^5
Listeria monocytogenes	PN-EN ISO 11290-1:2017-07, horizontal method	Chromogenic Listeria agar ISO 11290	was not found

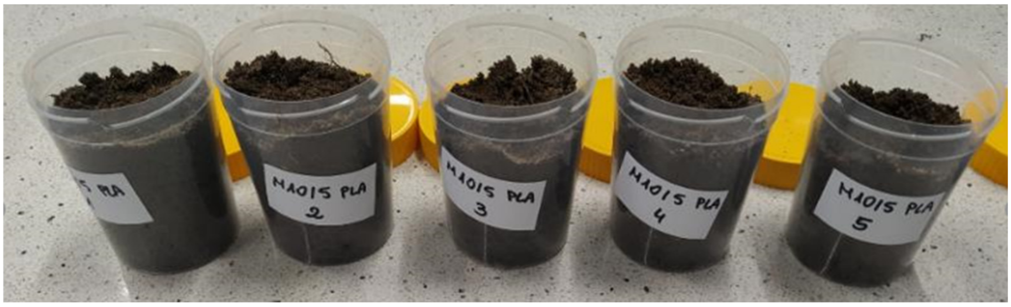

Figure S2. Containers with soil and PUR 10/5 + PLA samples prepared for placing in the incubation chamber.

Table S3. Images of samples at 4, 12 and 36 weeks of exposure to soil.

Sample	Incubation time [weeks]		
	4	12	36
PUR 10/5	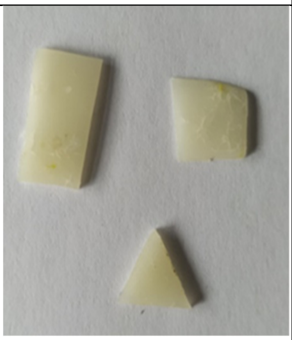	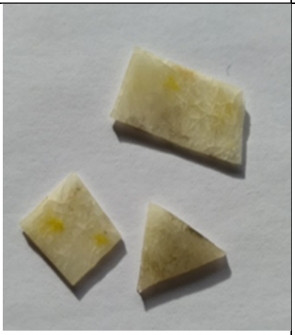	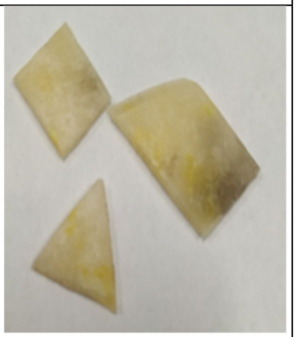

PUR 20/5+St	PUR 20/5+PLA	PUR 20/5	PUR 10/5+PLA
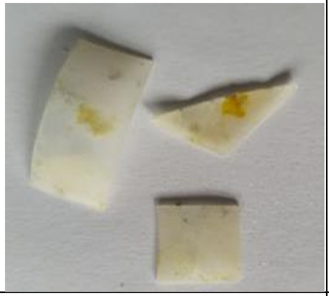	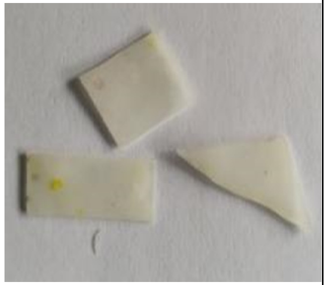	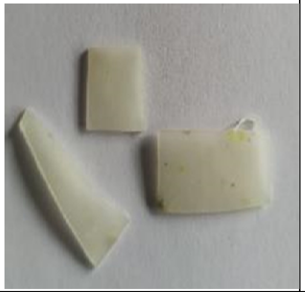	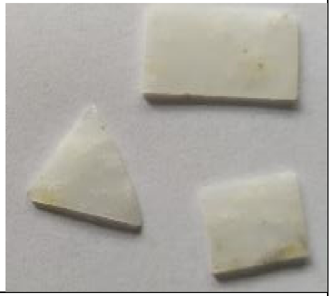
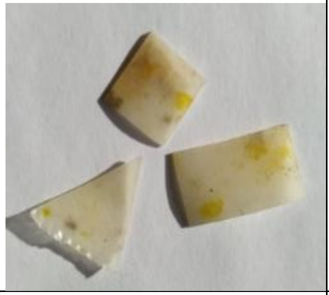	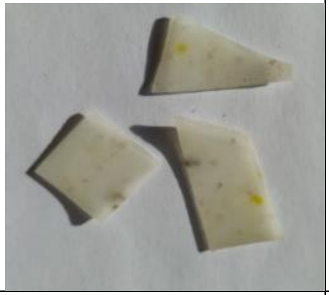	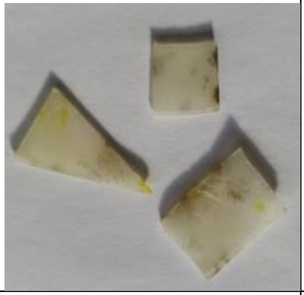	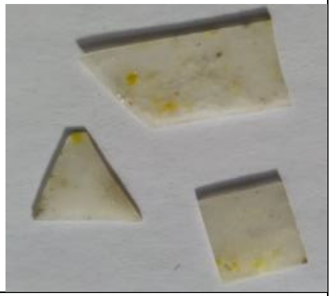
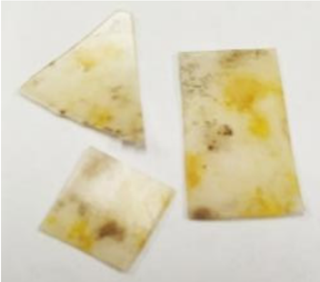	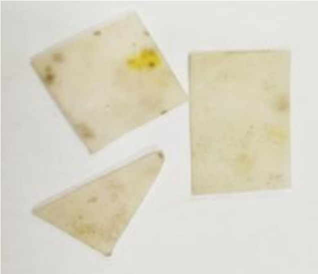	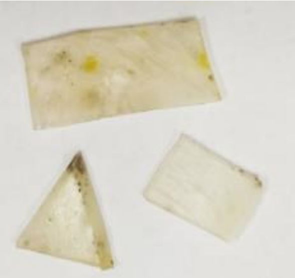	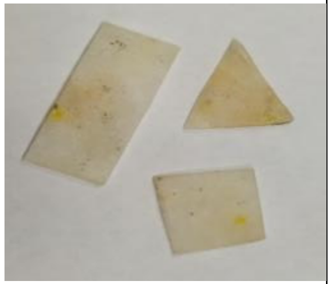

Table S4. The values of the wavenumbers of the bands corresponding to the vibrations of the bonds in groups potentially susceptible to hydrolysis before and after 36 weeks of exposure to soil.

**Sample**		ν(N-H) [cm^{-1}]	νC=O (ester) and Amide I [cm^{-1}]	Amide II δ(NH), ν(CN) [cm^{-1}]	Amide III ν(CN), δ(CO), ν(NH) [cm^{-1}]	ν(C=O)-O [cm^{-1}]	ν(C-O) (hydrogen bonded) [cm^{-1}]
**PUR10/5**	before	3369.0	1720.7	1523.0	1241.0	1187.5	1099.2; 1045.7
	after	3365.7	1721.2	1523.5	1240.0	1187.5	1097.3; 1044.7
**PUR 10/5+PLA**	before	3366.1	1721.2	1525.9	1240.0	1181.7	1085.7; 1045.2
	after	3356.5	1722.1	1525.4	1239.0	1180.2	1088.1; 1044.3
**PUR20/5**	before	3433.6	1722.1	1524.5	1240.0	1177.8	1099.2; 1046.2
	after	3344.4	1721.6	1524.5	1239.5	1177.3	1097.8; 1045.2
**PUR 20/5+PLA**	before	3369.5	1724.5	1523.0	1238.6	1180.7	1085.2; 1046.2
	after	3371.4	1723.1	1523.5	1239.0	1178.3	1096.8; 1045.2
**PUR 20/5+St**	before	3365.2	1722.6	1523.5	1239.5	1177.8	1100.2; 1045.7
	after	3357.0	1721.6	1524.0	1239.5	1178.3	1099.2; 1045.7

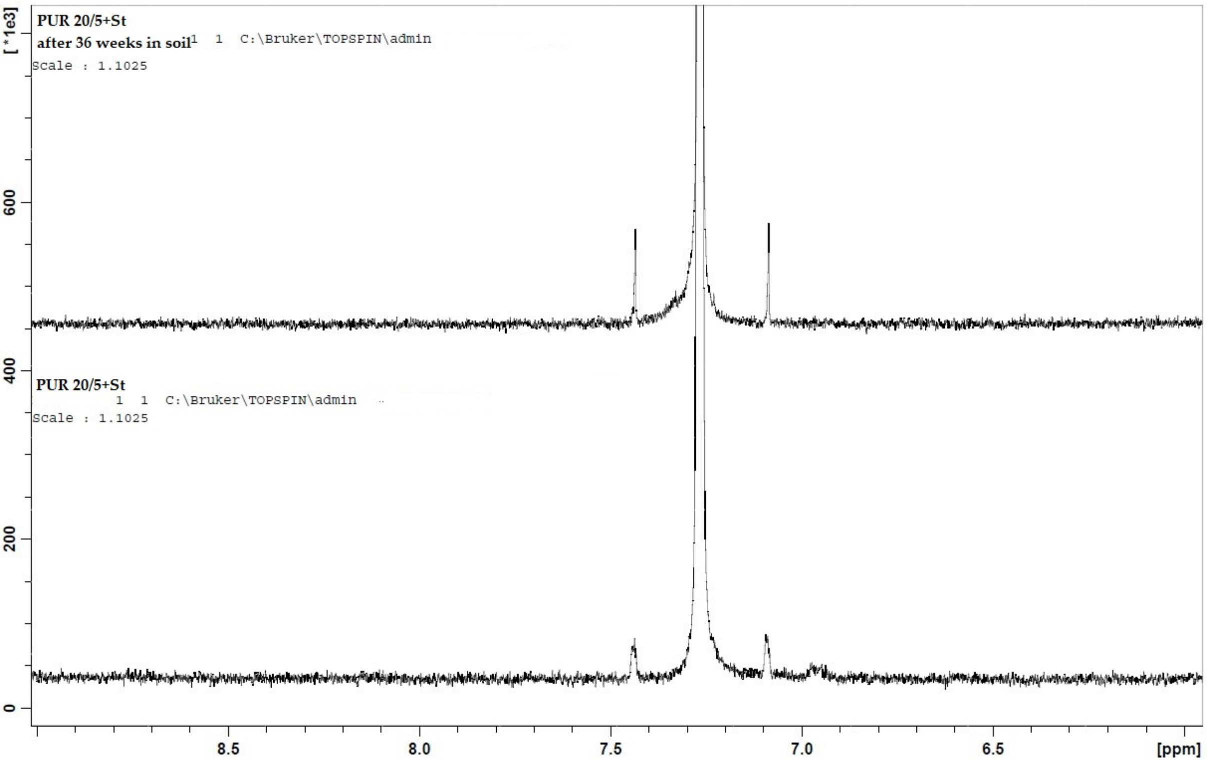

Figure S3. Range from 6 to 9 ppm of ^{1}HNMR spectra of PUR 20/5 + St before and after 36 weeks of exposure to soil.

Table S5. Microscopic images of the surface of PUR samples and their blends under reflected light before and after 36 weeks of exposure to soil.

Sample	Before incubation	After incubation
PUR 10/5	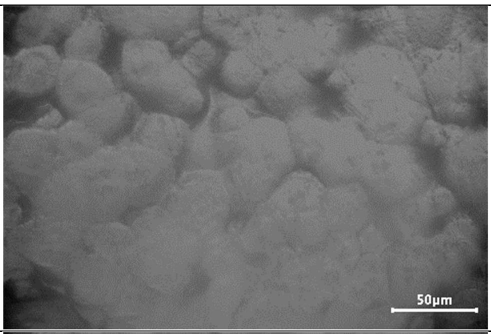	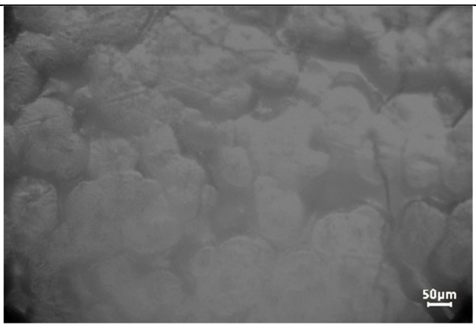
PUR 10/5<br>+ PLA	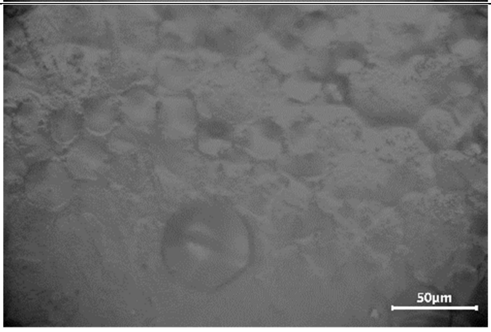	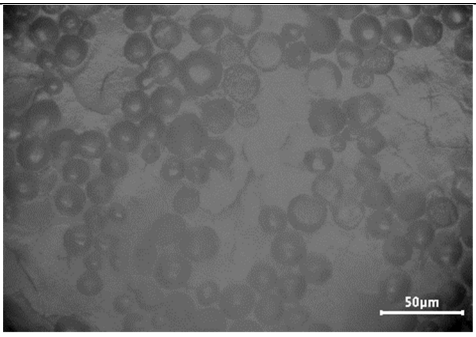
PUR 20/5	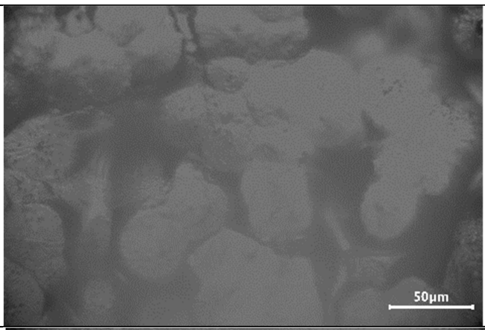	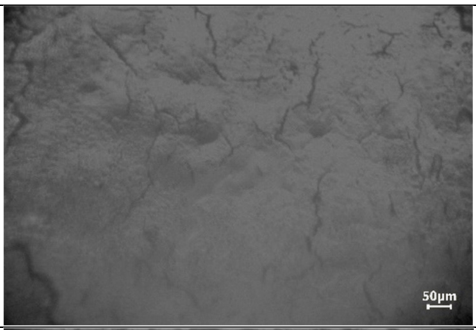
PUR 20/5<br>+ PLA	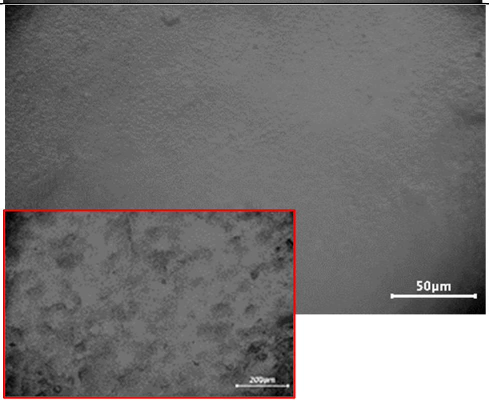 lower magnification	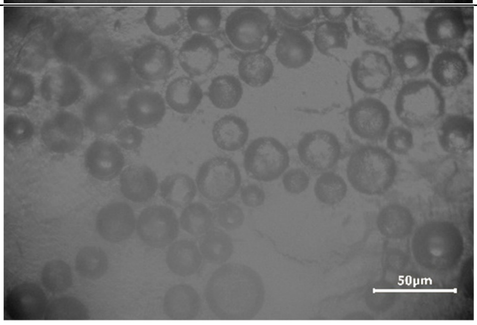
PUR 20/5<br>+<br>St	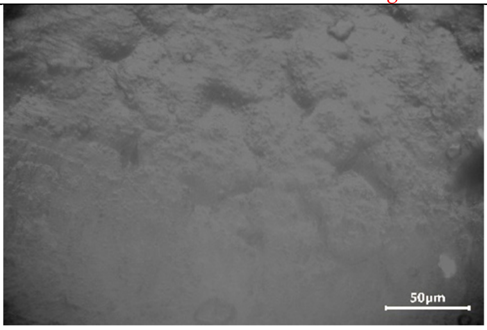	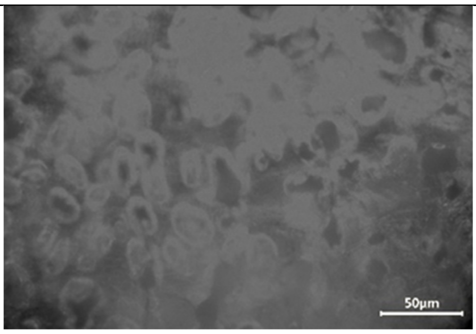

Table S6. Microscopic images of the surface of PUR samples and their blends under transmitted light before and after 36 weeks of exposure to soil.

**Sample**	**Before incubation**	**After incubation**
PUR 10/5	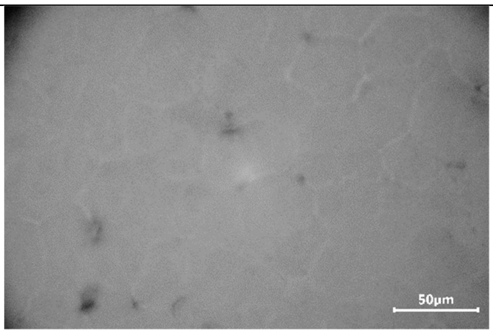	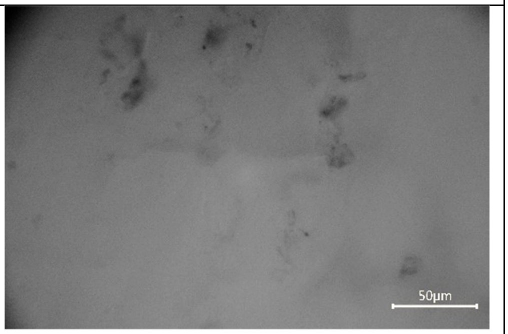
PUR 10/5<br>+ PLA	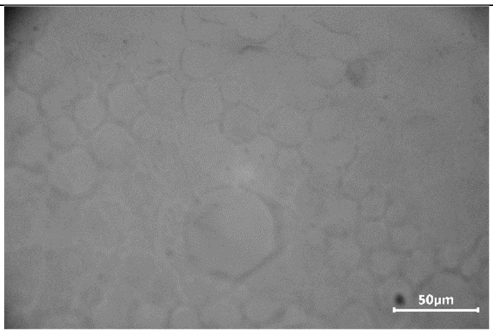	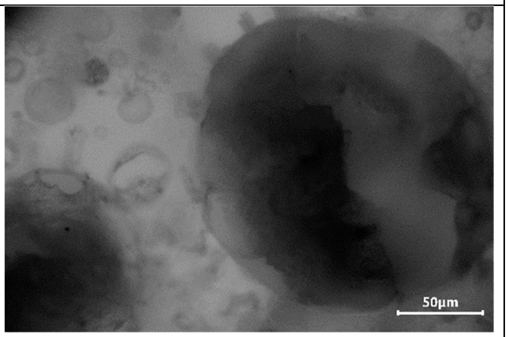
PUR 20/5	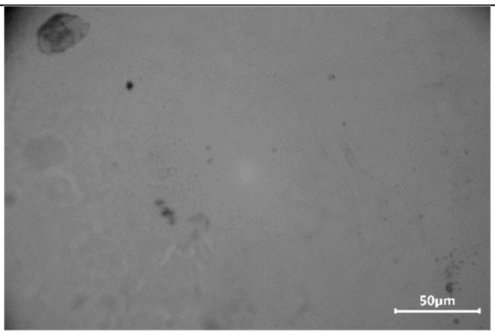	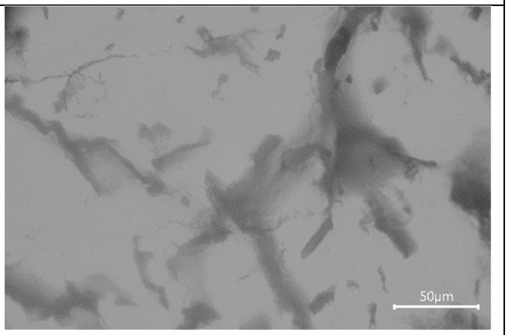
PUR 20/5<br>+ PLA	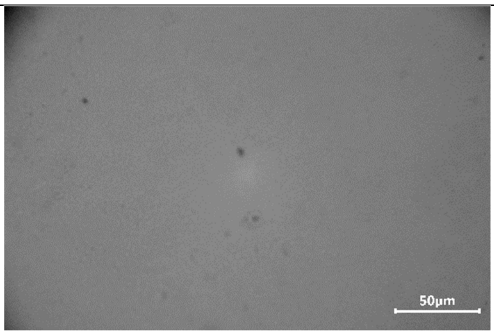	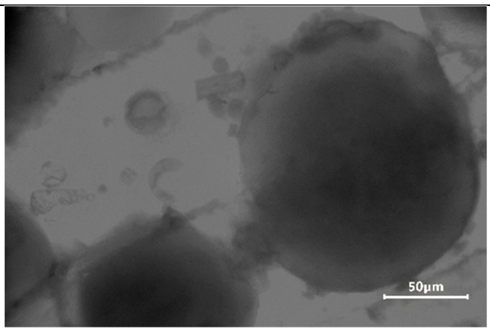
PUR 20/5<br>+ St	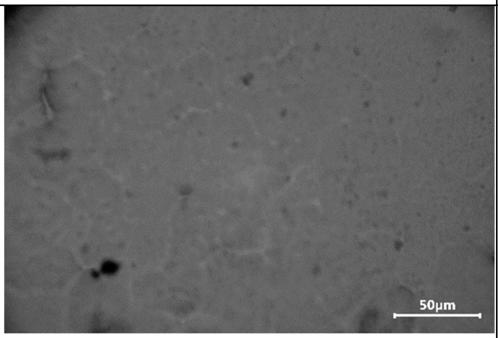	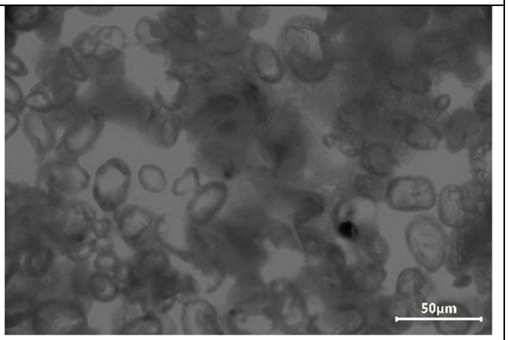

Table S7. The values of the contact angle of the pristine and degraded PURs and their blends, 0, 1 and 3 minutes after planting the drop on the surface

**Sample**	**Contact angle (± SD) [°] in time after immersion of drop on sample surface [min]**		
	0	1	3
PUR 10/5	75.7 (1.8)	69.9 (1.8)	64.7 (1.7)
PUR 10/5 S	65.2 (0.2)	58.5 (2.3)	51.4 (4.2)
PUR 10/5 + PLA	73.8 (1.7)	67.0 (1.1)	61.7 (1.3)
PUR 10/5 + PLA S	79.5 (2.2)	73.1 (1.4)	67.4 (1.2)
PUR 20/5	77.6 (1.0)	67.5 (1.9)	60.8 (1.6)
PUR 20/5 S	68.2 (0.2)	63.0 (0.4)	58.6 (0.6)
PUR 20/5 + PLA	72.8 (3.8)	65.1 (2.5)	60.0 (2.8)
PUR 20/5 + PLA S	90.8 (2.4)	82.4 (1.4)	74.4 (1.6)
PUR 20/5 + St	71.6 (5.8)	64.5 (5.4)	58.7 (5.3)
PUR 20/5 + St S	67.0 (4.7)	62.1 (3.6)	56.3 (2.8)
